# Five serum fatty acids are associated with subclinical hypothyroidism in a Chinese pregnant population

**DOI:** 10.1038/s41598-020-63513-7

**Published:** 2020-04-21

**Authors:** Ting Zhang, Yinyin Xia, Ting-Li Han, Hua Zhang, Philip N. Baker

**Affiliations:** 1 Department of Obstetrics and Gynaecology, The First Affiliated Hospital of Chongqing Medical University, Chongqing, 400016 China; 2 State Key Laboratory of Maternal and Fetal Medicine of Chongqing Municipality, Chongqing Medical University, Chongqing, 400016 China; 3 School of Public Health and Management, Chongqing Medical University, Chongqing, 400016 China; 4 Institute of Life Sciences, Chongqing Medical University, Chongqing, 400016 China; 5 Liggins Institute, The University of Auckland, Auckland, 1023 New Zealand; 6 College of Medicine, Biological Sciences and Psychology, University of Leicester, Leicester, UK

**Keywords:** Thyroid diseases, Thyroid diseases

## Abstract

Subclinical hypothyroidism (SCH) is a common endocrine disorder affecting women of reproductive age. Although SCH and abnormal fatty acid composition are often associated with adverse pregnancy outcomes and metabolic syndrome later in maternal and fetal life, the longitudinal relationship between SCH and serum fatty acids during pregnancy has rarely been studied. Therefore, the aim of this study was to investigate the association between SCH and maternal serum fatty acids throughout gestation. A total of 240 women enrolled in the Complex Lipids in Mothers and Babies (CLIMB) study in Chongqing, China were included in our study. Clinical information and maternal serum samples were collected at three time points during pregnancy: 11–14^th^, 22–28^th^, and 32–34^th^ weeks of gestation. Twenty serum fatty acids were quantified using gas chromatography-mass spectrometry (GC-MS) analysis. A majority of the 20 serum fatty acids increased as gestation progressed in women with a normal pregnancy and women experiencing SCH. Levels of arachidic acid, docosahexaenoic acid, and eicosenoic acid were significantly higher in the serum of women with SCH when compared to women with a normal pregnancy, in the second trimester. On the other hand, the levels of eicosadienoic acid and octadecanoic acid were significantly higher in SCH in the third trimester. Our findings demonstrate that serum fatty acid composition during the second and third trimesters was significantly associated with SCH in pregnant Chinese women.

## Introduction

Subclinical hypothyroidism (SCH) is a common endocrine disorder that can manifest in pregnant women. This complication of pregnancy is diagnosed when a mildly elevated serum thyroid-stimulating hormone (TSH) level above the upper limit of the trimester-specific reference is detected in combination with a normal free thyroxine (FT4) concentration during pregnancy^1,2^. SCH occurs in approximately 2–5% of all pregnant women^3–5^. In China, the incidence of SCH in pregnancy has reached epidemic proportions, affecting between 2.8% and 7.2% of all pregnancies^6–8^. SCH during pregnancy can lead to various maternal and fetal complications such as pregnancy loss, preterm birth, placental abruption, severe preeclampsia, gestational diabetes, and impaired offspring neurodevelopment^9–13^.

Several clinical studies have indicated that patients with SCH have altered serum lipid profiles and a higher prevalence of metabolic syndrome^14–16^. Elevated plasma free fatty acid (FFA) levels have been reported in patients with SCH regardless of age, sex, or menopausal status^14,17^. Fluctuations in fatty acid levels also have been associated with metabolic syndromes such as dyslipidemia, type-2 diabetes, and obesity^18^. Fatty acids play a crucial role in the support and regulation of a healthy pregnancy and normal infant development^19^. However, there is a lack of evidence directly relating the changes in serum fatty acids with SCH in pregnancy.

Considering the importance of fatty acids for both maternal and offspring health, as well as an increasing prevalence of SCH during pregnancy, our study aimed to investigate the changes in serum fatty acids in women with SCH, throughout pregnancy. We analyzed 20 fatty acids in maternal serum across the three trimesters of pregnancy in order to unravel their possible association with SCH for the identification of potential biomarkers for diagnosis or therapeutic intervention.

## Results

### Clinical characteristics

All clinical characteristics for the study population are listed in Table 1. Only the TSH levels were significantly higher in SCH women when compared to normal women (3.30 uIU/ml vs. 1.19 uIU/ml; *p* < 0.001). Other maternal characteristics including age, education, gravidity, parity, BMI, blood pressure, gestational age at sampling and FT4 exhibited no significant differences between case and control groups.

**Table 1:** Clinical characteristics of the study participants (n = 240).

	Normal (n = 222)	SCH (n = 18)	*P* value
Age, years	28 (26, 30)	28 (27, 31)	0.22
Total years of schooling	16 (15, 16)	16 (15, 16)	0.64
Gravidity	2 (1, 2)	2 (1, 2)	0.93
Parity	0 (0, 0)	0 (0, 0)	0.74
BMI (kg/m^2^)			
1^st^ trimester	20.6 (19.2, 22.4)	20.1 (18.5, 21.9)	0.35
2^nd^ trimester	22.9 (21.2, 24.8)	22.2 (20.6, 23.7)	0.30
3^rd^ trimester	24.5 (22.3, 26.2)	23.3 (22.2, 25.7)	0.51
sBP (mmHg)			
1^st^ trimester	112 (106.3, 121.0)	112 (110.0, 118.5)	0.76
2^nd^ trimester	116 (110.0, 122.0)	118 (112.0, 122.8)	0.65
3^rd^ trimester	116 (108.0, 120.0)	120 (110.5, 121.0)	0.30
dBP (mmHg)			
1^st^ trimester	70 (65.0, 76.0)	74.5 (70.0, 76.0)	0.17
2^nd^ trimester	71 (68.0, 76.0)	69.5 (66.3, 74.8)	0.29
3^rd^ trimester	72 (68.0, 76.0)	70.5 (63.5, 77.5)	0.84
GA at sampling (weeks)			
1^st^ trimester	12.7 (12.1, 13.3)	12.3 (12.1, 13.1)	0.23
2^nd^ trimester	24.3 (23.7, 24.6)	24.2 (23.9, 24.9)	0.47
3^rd^ trimester	32.3 (31.7, 32.7)	32.1 (23.9, 24.9)	0.23
s-TSH (uIU/ml)	1.19 (1.16, 1.39)	3.30 (3.16, 4.62)	1.41E-11***
FT4 (ng/ml)	0.83 (0.81, 0.86)	0.79 (0.75, 0.85)	0.29

All clinical data are summarized as median (25th percentile, 75th percentile) because each of the variables was not normally distributed. P values were calculated using a Mann-Whitney test, ***p < 0.001. Abbreviations: SCH, subclinical hypothyroidism; BMI, body mass index; BP, blood pressure; sBP, systolic blood pressure; dBP, diastolic blood pressure; GA, gestational age.

### Differences in the levels of serum fatty acids across trimesters

**Figure 1:**
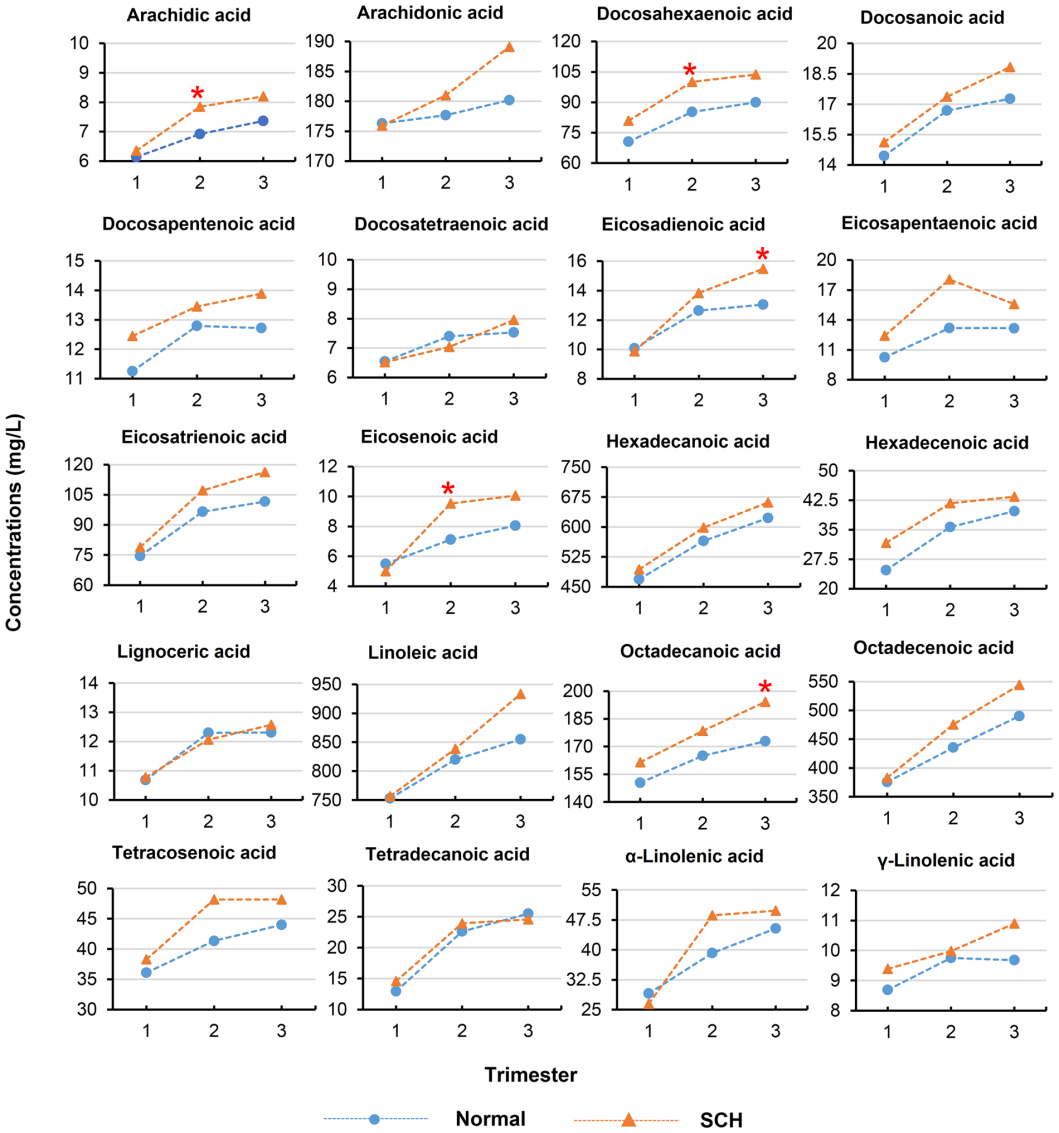
The concentrations of serum fatty acids in the first, second, and third trimesters collected from normal pregnancies and pregnancies with SCH. Red triangles represent serum fatty acid levels collected from women with SCH. Blue circles represent serum fatty acid levels from normal pregnancies. Red asterisks (*) indicate fatty acids with significantly different levels in SCH and normal pregnancies *(p*-values less than 0.05).

A total of 20 serum fatty acids were absolutely quantified in the maternal serum of the participants in their first, second, and third trimesters, as shown in Tables 2–4 and Fig. 1. Fatty acids generally increased in both SCH women and normal women as gestation progressed, particularly from the first to second trimester. However, this trend did not occur between the second and third trimesters. In contrast, eicosapentaenoic acid (EPA) was reduced in both SCH and normal groups from the second to the third trimester, while docosapentenoic acid (DPA) and γ-linolenic acid were only reduced in the normal women from the second to the third trimester.

**Table 2:** The concentrations, AUC, OR and statistical tests of maternal serum fatty acids between SCH and normal pregnancies in the first trimester.

Fatty acids	Fatty acids concentrations (mg/L)		AUC	OR (95%CI)	*P* value
	Normal (n = 222)	SCH (n = 18)			
Arachidic acid	6.14 ± 1.73	6.35 ± 1.93	0.48	1.07 (0.80,1.37)	0.63
Arachidonic acid	176.31 ± 52.16	175.96 ± 66.18	0.57	1.00 (0.99,1.01)	0.98
Docosahexaenoic acid	70.55 ± 22.35	80.98 ± 38.71	0.45	1.01 (1.00,1.03)	0.08
Docosanoic acid	14.45 ± 4.41	15.12 ± 5.53	0.49	1.03 (0.93,1.13)	0.54
Docosapentenoic acid	11.25 ± 4.43	12.45 ± 6.81	0.48	1.05 (0.95,1.14)	0.30
Docosatetraenoic acid	6.55 ± 2.31	6.52 ± 3.42	0.57	0.99 (0.79,1.19)	0.96
Eicosadienoic acid	10.08 ± 3.84	9.87 ± 4.58	0.54	0.99 (0.85,1.11)	0.82
Eicosapentaenoic acid	10.26 ± 8.51	12.42 ± 9.61	0.56	1.02 (0.97,1.06)	0.32
Eicosatrienoic acid	74.49 ± 33.57	78.80 ± 69.3	0.59	1.00 (0.99,1.01)	0.64
Eicosenoic acid	5.50 ± 2.41	5.00 ± 2.00	0.56	0.90 (0.68,1.11)	0.39
Hexadecanoic acid	469.15 ± 113.21	493.8 ± 167.37	0.47	1.00 (1.00,1.01)	0.39
Hexadecenoic acid	24.7 ± 12.69	31.63 ± 35.53	0.51	1.02 (0.99,1.04)	0.10
Lignoceric acid	10.68 ± 3.64	10.78 ± 5.06	0.54	1.01 (0.88,1.13)	0.92
Linoleic acid	752.88 ± 177.04	756.70 ± 187.03	0.53	1.00 (1.00,1.00)	0.93
Octadecanoic acid	150.47 ± 33.29	161.49 ± 42.01	0.57	1.01 (1.00,1.02)	0.19
Octadecenoic acid	375.63 ± 92.41	382.86 ± 134.38	0.53	1.00 (1.00,1.01)	0.76
Tetracosenoic acid	36.07 ± 13.14	38.28 ± 17.58	0.50	1.01 (0.98,1.04)	0.50
Tetradecanoic acid	12.96 ± 8.08	14.59 ± 14.60	0.53	1.02 (0.96,1.06)	0.45
α-Linolenic acid	29.09 ± 14.25	26.44 ± 13.41	0.58	0.98 (0.94,1.02)	0.44
γ-Linolenic acid	8.69 ± 5.96	9.39 ± 11.18	0.58	1.02 (0.94,1.08)	0.66

Data are presented as mean ± SD, *p < 0.05. Abbreviation: SCH, subclinical hypothyroidism; AUC, area under the curve; OR, odds ratios; CI, confidence intervals.

**Table 3:** The concentrations, AUC, OR and statistical tests of maternal serum fatty acids between SCH and normal pregnancies in the second trimester.

Fatty acids	Fatty acids concentrations (mg/L)		AUC	OR (95%CI)	*P* value
	Normal (n = 222)	SCH (n = 18)			
Arachidic acid	6.92 ± 1.74	7.85 ± 2.16	0.63	1.29 (1.01,1.65)	0.04 *
Arachidonic acid	177.67 ± 50.69	181.01 ± 53.4	0.50	1.00 (0.99,1.01)	0.79
Docosahexaenoic acid	85.31 ± 27.73	100.12 ± 38.51	0.61	1.01 (1.00,1.03)	0.04*
Docosanoic acid	16.69 ± 4.61	17.37 ± 4.76	0.53	1.03 (0.93,1.13)	0.55
Docosapentenoic acid	12.79 ± 4.74	13.45 ± 5.57	0.49	1.03 (0.93,1.13)	0.58
Docosatetraenoic acid	7.4 ± 2.33	7.04 ± 2.27	0.46	0.93 (0.73,1.15)	0.53
Eicosadienoic acid	12.65 ± 3.81	13.84 ± 4.22	0.57	1.07 (0.95,1.19)	0.21
Eicosapentaenoic acid	13.18 ± 8.84	18.07 ± 18.87	0.56	1.03 (1.00,1.07)	0.06
Eicosatrienoic acid	96.6 ± 35.11	107.19 ± 48.88	0.55	1.01 (0.99,1.02)	0.24
Eicosenoic acid	7.12 ± 3.72	9.54 ± 5.3	0.64	1.11 (1.01,1.21)	0.02*
Hexadecanoic acid	565.06 ± 127.01	598.47 ± 151.20	0.55	1.00 (1.00,1.01)	0.29
Hexadecenoic acid	35.64 ± 20.49	41.70 ± 28.66	0.56	1.01 (0.99,1.03)	0.25
Lignoceric acid	12.3 ± 4.22	12.07 ± 3.93	0.51	0.99 (0.87,1.10)	0.82
Linoleic acid	820.02 ± 168.63	838.34 ± 196.93	0.49	1.00 (1.00,1.00)	0.66
Octadecanoic acid	164.99 ± 32.94	178.49 ± 36.34	0.59	1.01 (1.00,1.02)	0.10
Octadecenoic acid	435.73 ± 99.58	475.67 ± 111.84	0.61	1.00 (1.00,1.01)	0.11
Tetracosenoic acid	41.34 ± 14.71	48.17 ± 17.93	0.60	1.02 (1.00,1.05)	0.07
Tetradecanoic acid	22.64 ± 11.28	23.93 ± 12.57	0.52	1.01 (0.97,1.05)	0.64
α-Linolenic acid	39.21 ± 19.59	48.67 ± 22.33	0.65	1.02 (1.00,1.04)	0.07
γ-Linolenic acid	9.75 ± 5.83	9.98 ± 8.94	0.54	1.01 (0.92,1.08)	0.88

Data are presented as mean ± SD, *p < 0.05. Abbreviation: SCH, subclinical hypothyroidism; AUC, area under the curve; OR, odds ratios; CI, confidence intervals.

**Table 4:** The concentrations, AUC, OR and statistical tests of maternal serum fatty acids between SCH and normal pregnancies in the third trimester.

Fatty acids	Fatty acids concentrations (mg/L)		AUC	OR (95%CI)	*P* value
	Normal (n = 222)	SCH (n = 18)			
Arachidic acid	7.37 ± 2.03	8.20 ± 1.79	0.64	1.19 (0.96,1.47)	0.10
Arachidonic acid	180.20 ± 52.59	189.09 ± 51.28	0.46	1.00 (0.99,1.01)	0.49
Docosahexaenoic acid	89.92 ± 28.57	103.73 ± 32.45	0.64	1.01 (1.00,1.03)	0.06
Docosanoic acid	17.27 ± 4.77	18.83 ± 5.92	0.57	1.06 (0.97,1.16)	0.19
Docosapentenoic acid	12.72 ± 4.70	13.89 ± 5.35	0.56	1.05 (0.95,1.15)	0.32
Docosatetraenoic acid	7.53 ± 2.39	7.96 ± 3.11	0.53	1.07 (0.88,1.27)	0.47
Eicosadienoic acid	13.05 ± 3.71	15.48 ± 5.34	0.64	1.15 (1.03,1.28)	0.01*
Eicosapentaenoic acid	13.17 ± 9.37	15.60 ± 10.57	0.59	1.02 (0.97,1.06)	0.30
Eicosatrienoic acid	101.58 ± 36.18	116.28 ± 45.63	0.58	1.01 (1.00,1.02)	0.11
Eicosenoic acid	8.06 ± 4.18	10.06 ± 4.03	0.67	1.08 (0.98,1.17)	0.08
Hexadecanoic acid	622.33 ± 139.05	661.60 ± 150.11	0.60	1.00 (1.00,1.01)	0.25
Hexadecenoic acid	39.69 ± 18.71	43.36 ± 20.89	0.56	1.01 (0.98,1.03)	0.43
Lignoceric acid	12.31 ± 4.05	12.57 ± 4.77	0.50	1.02 (0.90,1.13)	0.79
Linoleic acid	854.95 ± 173.94	933.57 ± 237.68	0.61	1.00 (1.00,1.01)	0.08
Octadecanoic acid	172.86 ± 33.15	194.13 ± 40.54	0.68	1.02 (1.00,1.03)	0.01*
Octadecenoic acid	489.96 ± 108.26	544.08 ± 137.12	0.61	1.00 (1.00,1.01)	0.05
Tetracosenoic acid	43.98 ± 17.17	48.20 ± 15.01	0.59	1.01 (0.99,1.04)	0.31
Tetradecanoic acid	25.52 ± 11.96	24.57 ± 11.16	0.52	0.99 (0.95,1.03)	0.74
α-Linolenic acid	45.39 ± 22.07	49.81 ± 20.76	0.57	1.01 (0.99,1.03)	0.41
γ-Linolenic acid	9.68 ± 5.90	10.90 ± 9.40	0.52	1.03 (0.95,1.10)	0.43

Data are presented as mean ± SD, *p < 0.05. Abbreviation: SCH, subclinical hypothyroidism; AUC, area under the curve; OR, odds ratios; CI, confidence intervals.

### Differences in the levels of serum fatty acids between women diagnosed with SCH and women with a normal pregnancy

**Figure 2:**
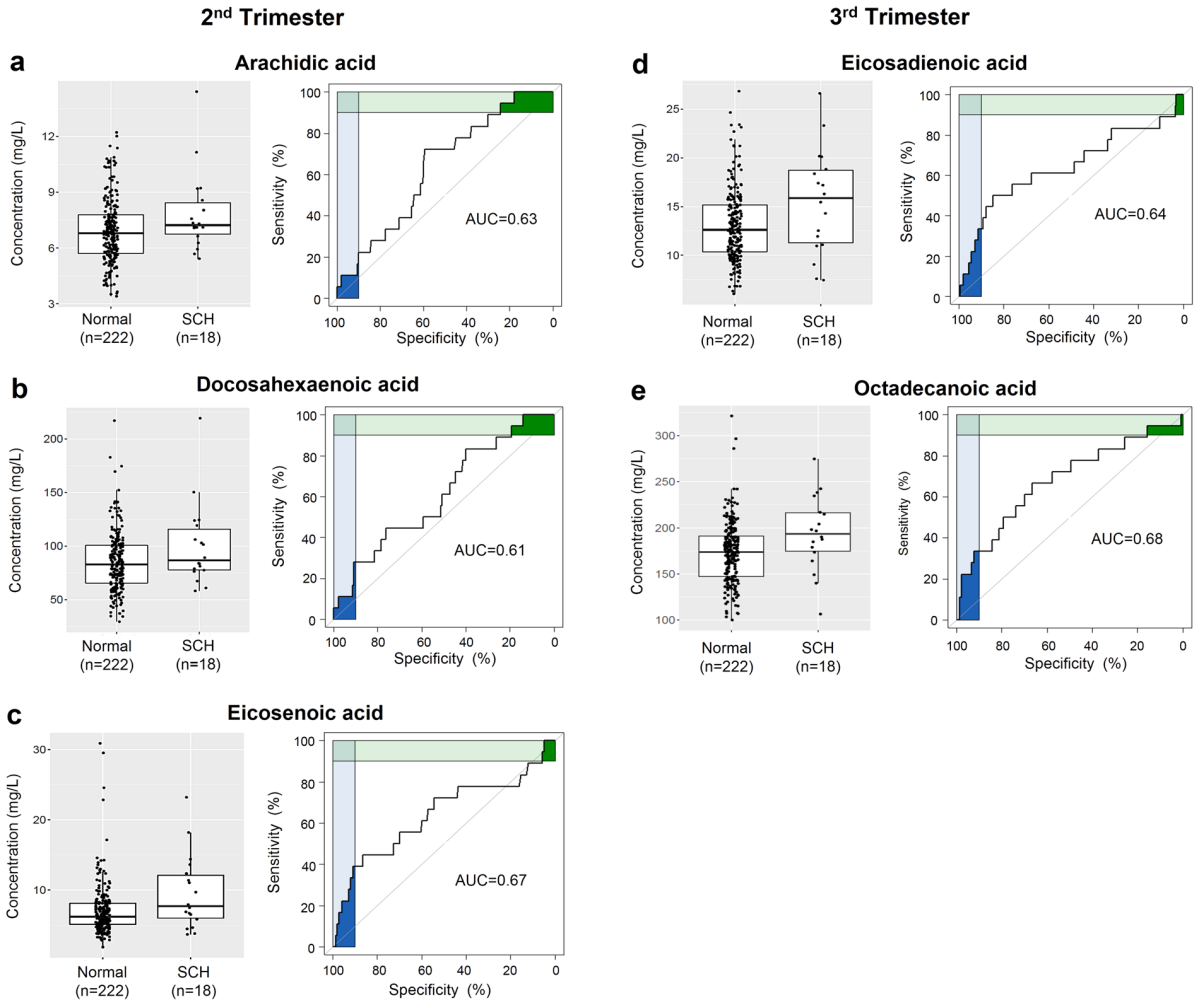
ROC curves for five fatty acids that were found to have significantly different levels in SCH and normal pregnancies.

Although the majority of serum fatty acids were detected at higher concentrations in SCH women compared to the normal women, none of the fatty acids in the first trimester significantly discriminated women diagnosed with SCH from normal groups (Table 2). Interestingly, we observed a significantly higher level of three fatty acids (arachidic acid, docosahexaenoic acid (DHA), and eicosenoic acid) in the SCH group in the second trimester, and two fatty acids (eicosadienoic acid and octadecanoic acid) in the third trimester. Among them, three out of the five fatty acids displayed odds ratios and their 95% confidence interval (CI) greater than 1 (Tables 3 and 4); arachidic acid (OR = 1.29, 95% CI 1.01 to 1.65), eicosenoic acid (OR = 1.11, 95% CI 1.01 to 1.21), and eicosadienoic acid (OR = 1.15, 95% CI 1.03 to 1.28). Meanwhile, the odds ratios calculated for DHA (OR = 1.01, 95% CI 1.00 to 1.03) and octadecanoic acid (OR = 1.02, 95% CI 1.00 to 1.03) were very close to 1.0. All five fatty acids had an area under the ROC curve between 0.61–0.68, as illustrated in Fig. 2.

## Discussion

To our knowledge, this is the first study to investigate the association between serum fatty acids and SCH among Chinese women across the three trimesters of pregnancy. In this study we observed that maternal serum fatty acid levels increased in both SCH and control groups as pregnancy progressed. In addition, we found a significantly higher concentration of three serum fatty acids (arachidic acid, DHA and eicosenoic acid) in women with SCH in the second trimester and higher concentration of another two serum fatty acids (eicosadienoic acid and octadecanoic acid) in the third trimester, when compared to normal pregnant women.

Our observations of increasing levels of serum fatty acids across the first two trimesters and to a lesser extent in the third trimester in both SCH and normal pregnant women, have been found previously in studies based on normal pregnancy^20–23^: Al *et al*.^20^ analyzed plasma essential fatty acids (EFA) at eleven different time points during pregnancy (at 10, 14, 18, 22, 26, 30, 32, 34, 36, 38 and 40 weeks) in 110 healthy pregnant women in the Netherlands; Otto *et al*.^21^ evaluated plasma EFA at three different periods (before 18 weeks and at 22 and 32 weeks of gestation) in 239 healthy pregnant women from the Netherlands, Hungary, Finland, England, and Ecuador; Stewart *et al*.^22^ assessed the fatty acid composition of the erythrocyte membranes in each trimester of pregnancy (mean gestational weeks of 12.5, 26.1 and 35.5) in 47 pregnant Australian women without any metabolic complications; and Pinto *et al*.^23^ examined serum fatty acid concentrations in the 5–13, 20–26 and 30–36 weeks of gestation in 146 healthy pregnancies in Brazil. The possible underlying reason for all of the described studies to have observed a similar increasing trend of fatty acid levels from the first to the second trimester, may be due to the accumulation of maternal fat reserves that occurs to meet the anabolic storage phase in the early and middle periods of gestation. This phenomenon is mediated by the progressive increase in insulin level, promoting lipogenesis and suppression of lipolysis via progesterone and cortisol^24,25^. Meanwhile, the observed trend of a lesser increase or sustained levels of fatty acids in the third trimester may occur as a result of upregulated lipolysis in maternal fat depots. The subsequent stimulation of the lipid transportation across the placenta to meet the increased energy demands of the mother and the increased fetal growth rate^26–28^.

Not only were the majority of the fatty acid levels raised as pregnancy progressed in both SCH and normal participants, we found that women diagnosed with SCH generally demonstrated a higher concentration of fatty acids than the normal pregnancies. An earlier study reported that TSH was the dominating lipolytic hormone *in vitro* during the neonatal period, when serum TSH levels surge dramatically^29^. FFAs have been found previously to be higher in SCH patients and the higher blood glycerol levels observed suggested that this increase in circulating FFAs was, at least in part, caused by enhanced lipolysis^17^. Similarly, another study showed that TSH raised serum FFA levels i*n vivo* by stimulating adipocyte lipolysis, managed by phosphorylation of perilipin and hormone-sensitive lipase in a protein kinase A-dependent manner in differentiated adipocytes^30^. These studies suggest that TSH may exert extrathyroidal effects to increase FFAs by elevating lipolysis, therefore playing an important regulatory role in lipolysis during pregnancy. Furthermore, there were five long-chain fatty acids, namely arachidic acid, DHA, eicosenoic acid, eicosadienoic acid, and octadecanoic acid, which were significantly higher in the serum of women diagnosed with SCH in pregnancy, in either the second or third trimester. It has previously been reported that patients with hypothyroidism had a higher level of octadecanoic acid in their serum lipid profile^31^. In a rat model of hypothyroidism, an increased level of DHA was found in the liver^32^. Therefore, these five long-chain fatty acids may have the potential to discriminate SCH from normal pregnancy in the middle and later stages of pregnancy. However, caution is advised when interpreting the significance of DHA and octadecanoic acid in relation to SCH because their odds ratios were very close to 1.

Our research has several limitations that deserve mention. Firstly, the sample size of the SCH group was small in this study. There were only 18 pregnant women who met the selection criteria for SCH, without other pregnancy complications. Secondly, maternal dietary intake data should be included to evaluate how maternal diets were linked to the serum fatty acid levels. Lastly, all our subjects diagnosed with SCH were administrated levothyroxine (LT4) treatment, which may interfere with the serum fatty acid outcomes. Nevertheless, some meta-analyses investigating the effect of LT4 treatment on lipid profiles in SCH patients concluded that LT4 replacement did not significantly affect triglyceride levels^33–35^.

In conclusion, our study has highlighted the association between SCH and maternal serum fatty acid profiles and shortlisted five fatty acids (arachidic acid, DHA, eicosenoic acid, eicosadienoic acid, and octadecanoic acid) that were significantly increased in women diagnosed with SCH during pregnancy. Future studies should consider validating our findings using a larger SCH sample size as well as animal models to further understand the pathophysiological mechanisms underlying the link between fatty acids and SCH during pregnancy.

## Methods

### Study participants

**Figure 3:**
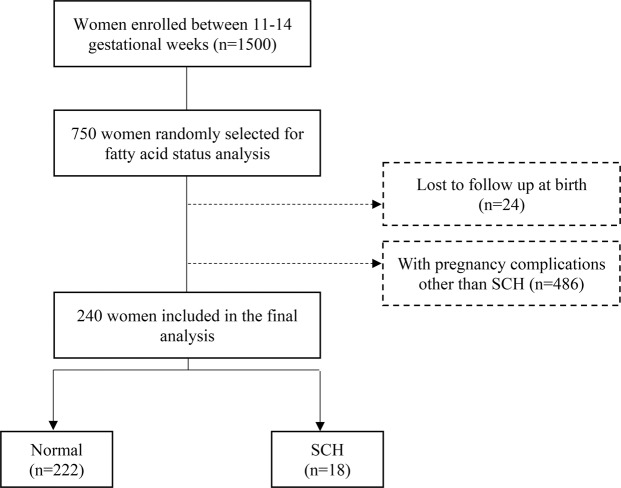
Flowchart of study participants.

All participants were selected from the Complex Lipids in Mothers and Babies (CLIMB) study. The CLIMB study was conducted at the First Affiliated Hospital of Chongqing Medical University and Chongqing Health Centre for Women and Children in China from September 2015 to June 2017. The details of the CLIMB study have been published previously^36^. A total of 1,500 women were recruited into the CLIMB study. For fatty acids analysis, 750 women were selected at random using randomly generated numbers. We excluded women lost to follow up at birth (n = 24) and those who had other adverse pregnancy outcomes (n = 486) such as gestational diabetes mellitus (GDM), and pregnancy-induced hypertension (PIH). This resulted in 240 women who were eligible for inclusion in the study; 1) 222 normal women without any pregnancy complications and 2) 18 women who were diagnosed with SCH based on the criteria used in China (Supplementary Table 1) which included having TSH > the upper limit of reference during pregnancy (97.5th percentiles) and a normal FT4. A flowchart of the study participants included in this study is shown in Fig. 3. All procedures performed in this study were in accordance with the principles in the Declaration of Helsinki 1964 and the International Conference on Harmonisation Good Clinical Practice E6 (ICH-GCP). The study was approved by the Ethics Committee of Chongqing Medical University (2014034). Written informed consent was obtained from all participants included in the study at enrollment.

### Clinical information and sample collection

Clinical information, demographic factors (maternal age, total years of schooling, last menstrual period (LMP), gravidity, parity), gynecologic and obstetric history (gravidity, delivery, abortion, infertility) were collected at enrollment. Maternal anthropometry (body mass index (BMI) and blood pressure (BP)) and maternal blood samples were collected at three visits during pregnancy (11–14, 22–28, and 32–34 gestational weeks) by trained nurses. Maternal blood samples were collected from participants in the morning after an overnight fast. Fasted blood samples were collected from the antecubital vein into vacutainer tubes containing separator gel and separated by centrifugation twice (3000 rpm at 4 °C for 10 min, then 4000 rpm at 4 °C for another 10 min). The supernatant was transferred into cryotubes (Micronic, Lelystad, The Netherlands) then stored at −80 °C until further processing. Pregnancy outcomes were diagnosed and managed by experienced obstetricians. Throughout the pregnancy, maternal gestational week was determined by LMP and mid-trimester ultrasound information (if the two estimates differed by more than seven days, gestational age was based on the obstetric ultrasound data).

### Reagents and fatty acid standards

Twenty one fatty acid standards were purchased from Sigma-Aldrich (St. Louis, MO, USA) (Supplementary Table 2). Methanol (chromatography grade) and n-hexane (chromatography grade) were purchased from Merck-Chemicals, KGaA (Germany). Hydrochloric acid and Milli-Q pure water were purchased from Guangzhou Chemical Reagent Factory (Guangzhou, China). Stock solutions of the 20 fatty acids and the internal standard (heptadecanoic acid) were prepared at 250 mg/mL in n-hexane. Working solutions were made up with methanol at concentrations of 0.10, 0.50, 2.5, 25, 100, 250 mg/L. All standard solutions were stored at −20 °C until required. The internal standard working solution was a 20 mg/L heptadecanoic acid n-hexane solution.

### Sample preparation and gas chromatography-mass spectrometry (GC-MS) analysis

A 250 uL aliquot of thawed serum from each participant was spiked with 250 uL of the internal standard (IS) working solution, followed by the addition of 1 mL hydrochloric acid/methanol and a mix using a vortex mixer. The sample was then incubated at 90 °C for 3 h using an electric blast drying oven (Shanghai Yiheng Scientific Instrument Co., Ltd.). After cooling, 2 mL of n-hexane was added to extract the fatty acid methyl ester products and the sample was vortexed. The supernatant was isolated after centrifugation and then evaporated to dryness under nitrogen. Following the addition of 400 uL n-hexane to dissolve the fatty acid methyl esterification product, the suspension was mixed and then transferred to a sample vial (Agilent Technologies, USA) prior to mass spectrometry analysis.

Gas Chromatography-Mass Spectrometry (GC-MS) was performed using an Agilent 7890B gas chromatograph coupled to a 5977 A mass spectrometer (Agilent Technologies, USA). The fatty acid separation was performed on the DB-23 capillary column (20 m × 0.18 mm × 0.20 μm, Agilent Technologies, USA). Helium was used as the carrier gas with a constant flow rate of 0.78 ml/min. The 1 μL of derivatized sample was injected into the inlet set at 230 °C, using the splitless inlet mode. Free fatty acid methyl esters were separated using the following oven temperature program: (1) 50 °C hold for 1 min; (2) increased to 175 °C at 25 °C/min; (3) reached 223 °C at 4 °C/min; and (4) hold at 223 °C for 8 min. The ion-trap mass spectrometer was operated in full scan (Scan) and selected ion scan (SIM) monitoring mode (mass range: 60.00–450.00 m/z). The transfer line was maintained at 230 °C, the source temperature was set at 220 °C, and solvent delay time was set at 10 min.

### Fatty acid quantification and statistical analysis

The chromatographic height of each of the fatty acids was extracted using Agilent ChemStation (version 2.6). Levels of individual serum fatty acids were first normalized by the internal standard and then quantified to absolute concentration using calibration curves derived from the corresponding chemical standard. A student’s t-test and non-parametric Mann-Whitney U test were executed in R to compare clinical characteristics between women with a normal pregnancy and women diagnosed with SCH. Prior to metabolomic statistical analysis, all fatty acid concentrations were corrected to a Gaussian distribution through logarithmic transformation and Pareto scaling. The area under the receiver operating characteristic (ROC) curve was analysed using the pROC R-package^37^. Odds ratios (OR) were calculated to assess associations between the levels of fatty acids and the occurrence of SCH. Tukey’s HSD test was implicated to account for multiple comparisons. A *p*-value <0.05 was considered statistically significant. Data were described as mean ± SD or median (IQR) for continuous variables. Boxplots were illustrated using ggplot2 R-based packages^38^.

## Supplementary information

Supplementary material.

## Data Availability

The datasets generated and/or analyzed during the current study are available from the corresponding author on reasonable request.
