# Supplementary material for: Five serum fatty acids are associated with subclinical hypothyroidism in a Chinese pregnant population
Source: Sci Rep. 2020 Apr 21;10:6743. doi: 10.1038/s41598-020-63513-7 (PMC7174292; doi:10.1038/s41598-020-63513-7)
Supplement: Supplementary file 1 — Supplementary material. [file 41598_2020_63513_MOESM1_ESM.pdf]

# Five serum fatty acids are associated with subclinical hypothyroidism in a Chinese pregnant population

Ting Zhang[1,2*], Yinyin Xia[3*], Ting-Li Han[1,4,5#], Hua Zhang[1,2#], Philip N Baker[6]

**Supplementary Table 1**. Reference ranges for free thyroxine (FT4) and thyroid-stimulating hormone (s-TSH) during pregnancy in Chongqing, China (Endocrine Laboratory, First Affiliated Hospital of Chongqing Medical University).

| | FT4 (ng/ml) | s-TSH (uIU/ml) |
|---|---|---|
| First trimester | 0.63-1.23 | 0.13-3.99 |
| Second trimester | 0.44-0.82 | 0.47-4.86 |
| Third trimester | 0.44-0.80 | 0.58-5.51 |

**Supplementary Table 2.** Standard and internal standard substance information.

| FAME standards* | Fatty acids | Oders | Manufacturer | Concentration | Batch number |
|---|---|---|---|---|---|
| Methyl myristate | Tetradecanoic acid (C14:0) | 1 | Sigma | ≥99% | Lot#071M1509V |
| Methyl palmitate | Hexadecanoic acid (C16:0) | 2 | Sigma | ≥99% | Lot#SLBG4424V |
| Methyl palmitoleate | Hexadecenoic acid (C16:1n7) | 3 | Sigma | ≥99% | Lot#SLBB1046V |
| Heptadecanoic acid | Heptadecanoic acid (C17:0) | 4 | Sigma | ≥99% | Lot 099K1181 |
| Methyl stearate | Octadecanoic acid (C18:0) | 5 | Sigma | ≥99% | Lot#BCBK6253V |
| Methyl oleate | Octadecenoic acid (C18:ln9) | 6 | Sigma | ≥99% | Lot#MKBL9030V |
| Methyl linoleate | Linoleic acid (C18:2n6) | 7 | Sigma | ≥99% | Lot#BCBG7530V |
| Methyl-γ-linolenate | γ-Linolenic acid (C18:3n6) | 8 | Sigma | ≥99% | Lot#SLBD0092V |
| α-Methyl linolenate | α-Linolenic acid (C18:3n3) | 9 | Sigma | ≥99% | Lot#SLBD5909V |
| Methyl arachidate | Arachidic acid (C20:0) | 10 | Sigma | ≥99% | Lot#SLBD2753V |
| Methyl cis-11-eicosenoate | Eicosenoic acid (C20:ln9) | 11 | Sigma | ≥99% | Lot#SLBF1161V |
| cis-11,14-Eicosadienoic acid, methyl ester | Eicosadienoic acid（C20:2n6） | 12 | Sigma | ≥99% | 1G010010 |
| 8,11,14-Eicosatfienoic acid, methyl ester | Eicosatrienoic acid (C20:3n6) | 13 | Sigma | ≥99% | 4G010010 |
| cis-5,8,11,14-Eicosatetraenoic acid, methyl ester | Arachidonic acid (C20:4n6) | 14 | Sigma | ≥99% | Lot#SLBC5923V |
| Docosanoic acid, methyl ester | Docosanoic acid (C22:0) | 15 | Sigma | ≥99% | Lot#80207 |
| Methyl all-cis-5,8,11,14,17-eicosapentaenoate | Eicosapentaenoic acid (C20:5n3) | 16 | Sigma | ≥99% | Lot#BCBK2894V |
| cis-7,10,13,16-Docosatetranoic acid, methyl ester | Docosatetraenoic acid (C22:4n6) | 17 | Sigma | ≥99% | Lot#SLBG5166V |
| Tetracosanoic acid, methyl ester | Lignoceric acid (C24:0) | 18 | Sigma | ≥99% | Lot#00818 |
| Methyl all-cis-7,10,13,16,19-docosapentaenoate | Docosapentenoic acid (C22:5n3) | 19 | Sigma | ≥99% | Lot#BCBF6777V |
| Methyl 4,7,10,13,16,19-docosahexaenoate | Docosahexaenoic acid (C22:6n3) | 20 | Sigma | ≥99% | Lot#BCBL9396V |
| Methyl cis-15-tetracosenoate | Tetracosenoic acid (C24:1n9) | 21 | Sigma | ≥99% | Lot#BCBK2334V |

Abbreviation: FAME, fatty acid methyl ester
